# Flooding and fragment size interact to determine survival and regrowth after fragmentation in two stoloniferous *Trifolium* species

**DOI:** 10.1093/aobpla/plu024

**Published:** 2014-05-22

**Authors:** Heidrun Huber, Eric J. W. Visser, Gijs Clements, Janny L. Peters

**Affiliations:** 1 Department of Experimental Plant Ecology, Institute for Water and Wetland Research, Radboud University Nijmegen, Heyendaalseweg 135, 6525 AJ Nijmegen, The Netherlands; 2 Department of Molecular Plant Physiology, Institute for Water and Wetland Research, Radboud University Nijmegen, Heyendaalseweg 135, 6525 AJ Nijmegen, The Netherlands

**Keywords:** Clonal growth, disturbance, fragmentation, soil flooding, *Trifolium fragiferum*, *Trifolium repens*.

## Abstract

Clonal plants, which reproduce by means of stolons and rhizomes, are common in frequently flooded habitats. Resilience to disturbance is an important trait enabling plants to survive in such highly disturbed habitats. Resource storage is thought to enable clonal plants to resume growth after clonal fragmentation caused by disturbance. Here we investigated if submergence prior to disturbance reduces survival and regrowth of clonal fragments and whether or not genotypes originating from highly disturbed riverine habitats are more resistant to mechanical disturbance than genotypes from less disturbed coastal dune slack habitats. We further tested if variation in survival and regrowth was affected by internode size. Clones from contrasting habitats of two closely related *Trifolium* species were first genotypically characterized by amplification fragment length polymorphism and then subjected to soil flooding and subsequent clonal fragmentation. These species differ with respect to their abundance in riverine and dune slack habitats, with *Trifolium repens* mainly occurring in riverine grasslands and *Trifolium fragiferum* in coastal dune slacks. Soil flooding decreased survival and regrowth by up to 80 %. Plants originating from riverine grasslands were less negatively affected by fragmentation than plants from dune slack habitats. Surprisingly, ramets did not always benefit from being attached to a larger internode, as internode size was often negatively correlated with survival after fragmentation. Regrowth, on the other hand, was generally positively correlated with internode size. These unexpected results indicate that there may be contrasting selection pressures on internode size in stoloniferous species growing in severely disturbed habitats.

## Abstract (precis)

Clonal plants are common in frequently flooded habitats because of their resilience to disturbance. We investigated if submergence prior to fragmentation of clones of two clover species reduced survival and regrowth of clonal fragments, and if these fitness parameters were different between genotypes from highly disturbed river forelands and less disturbed coastal dune slacks. We found that soil flooding severely decreased survival and regrowth, and that plants from the more disturbance-prone habitat were less negatively affected by fragmentation. However, internode size was, surprisingly, often negatively correlated with survival after fragmentation, but positively correlated with regrowth. Apparently, contrasting selection pressures exist on internode size in stoloniferous species growing in disturbed habitats.

## Introduction

Flooding is an increasingly common stress affecting plant growth and vegetation composition in flood-prone areas. Recent global change scenarios predict that the incidence of flooding events will increase in the coming decades due to changes in rainfall patterns (Knapp *et al.* 2008). Flooding affects plant growth and survival in various ways, as it is a compound stress (Bailey-Serres and Voesenek 2008). Oxygen deficiency, light limitation, toxic soil compounds and mechanical disturbance may all act at the same time during flooding. These stresses may each select for specific plant traits, but it is remarkable that plant communities in flood-prone habitats are often characterized by a high prevalence of vegetatively reproducing plants (Benot *et al.* 2011), i.e. forming stolons, rhizomes or root suckers (Fischer and Van Kleunen 2001; Klimesova and de Bello 2009). Stolons and rhizomes play an important role in transport of resources between connected ramets (Watson 1986; Landa *et al.* 1992; Van Kleunen *et al.* 2000), and also have an important storage function (Suzuki and Stuefer 1999; Watson 2008). It has been argued that the presence of parts of the stolon can positively affect plant survival after clone fragmentation (Stuefer and Huber 1999; Dong *et al.* 2010, 2011, 2012; Song *et al.* 2013). This may be beneficial after a flooding event when part of the clone has become disconnected due to mechanical stress as a result of strong water currents or mortality of the remainder of the clone. Clonal species can quickly resprout from dormant meristems after clone fragmentation, by reallocating resources stored in the stolons and rhizomes to the sites of new leaf and root production (Klimesova and Klimes 2007). Apart from the negative effects of flooding-induced disturbance, flooding may also play an important role as a long-distance dispersal agent (Korpelainen *et al.* 2013). Clonal fragments may be spread along the riverine systems during flooding events, thereby allowing plants to invade new habitats or already established populations. Survival after clone fragmentation is therefore not only important for the maintenance of existing populations, but may also have important consequences for gene flow among populations or establishment of new populations.

The ability to regrow after clone fragmentation may depend on the environmental conditions (Puijalon *et al.* 2008; Bornette and Puijalon 2011). Moreover, different stresses may interact and the conditions experienced earlier may affect responsiveness to subsequent stress factors (Weinig and Delph 2001; Huber *et al.* 2012; von Wettberg *et al.* 2012). Stress events may precondition plants and prime them to respond to subsequent stresses, even if the stresses are of different nature (Bruce *et al.* 2007). It has, for example, been shown that both limited nutrient availability and mechanical stress caused by high water velocity increased plant survival after fragmentation in the aquatic species *Berula erecta* (Puijalon and Bornette 2006; Puijalon *et al.* 2008). A general response of increased allocation to clonal structures in plants experiencing environmental stress may explain this result. However, repeated environmental stress may also reduce plant growth and survival. This can especially occur if environmental stress negatively affects storage, either by reducing allocation to storage due to limited resource uptake or by depleting previously stored resources (e.g. caused by investment in increased elongation (Huber *et al.* 2012; Xu *et al.* 2013). The direction and nature of the effects of prior stresses on the response to subsequent fragmentation, however, largely remain unclear.

Plants have evolved different mechanisms to alleviate the negative effects of flooding events. Translocation of resources to leaf growth, production of adventitious roots, aerenchyma formation and elongation of petioles or leaf tissues may enable plants to restore oxygen supply (Visser and Voesenek 2005; Bailey-Serres and Voesenek 2008; Colmer and Voesenek 2009). It has been shown that the ability of plant species to express these adaptations strongly differs along flooding gradients (Visser *et al.* 1996; Voesenek *et al.* 2004; Banach *et al.* 2009). Strong selection pressures driven by a different frequency or likelihood of flooding have resulted in a distinctly different set of species within a narrow geographical range (van Eck *et al.* 2004). Also within flood-prone habitats, different types and frequencies of flooding may impose distinctly different selection pressures on plants (Voesenek *et al.* 2004; Chen *et al.* 2011). Coastal dune slacks and riverine grasslands are both prone to flooding. However, both habitat types are exposed to different flooding regimes, with rapidly rising water levels and strong currents during the growing period of plants in riverine grasslands, as opposed to mainly winter and early spring flooding with a much more gradual rise of the water level and no streaming water in coastal dune slacks. It is likely that plants in these contrasting flood-prone areas have evolved different mechanisms to cope with the flooding stress. In this manuscript we ask to what extent prior flooding affects the ability of plants to survive and regrow after mechanical disturbance, and whether different flooding regimes have led to the selection of locally adapted ecotypes that differ in their responses to fragmentation after flooding. Accordingly, we performed an experiment with the two closely related stoloniferous clover species *Trifolium repens* and *T. fragiferum* to test the following hypothesis: (i) As commonly observed in other species, internode size was expected to be positively linked to survival and regrowth after fragmentation. (ii) Previous flooding was expected to affect survival and regrowth after fragmentation, either positively by preconditioning of plant responses to further fragmentation, or negatively due to reserve depletion and negative effects of internode size. (iii) As *T. repens* often occurs in disturbed riverine habitats and *T. fragiferum* favours less disturbed dune slack grasslands, we expected *T. fragiferum* to be less tolerant to fragmentation following a flooding episode than *T. repens*. (iv) It was anticipated for both species that genotypes originating from the disturbance-prone river floodplains would be more disturbance tolerant than genotypes originating from the more sheltered dune slack environments.

## Methods

### Species and habitat description

Two closely related stoloniferous clover species *T. repens* and *T. fragiferum* were used for the experiments. These species have a similar developmental pattern: they both grow by means of aboveground stolons composed of ramets and each ramet consists of one internode, one leaf, one meristem located in the leaf axil (potentially differentiating into a flower or a side branch) and root primordia (Huber and Wiggerman 1997; Huber and During 2000; Huber *et al.* 2009). The species differ, however, in their ecological niches. While *T. fragiferum* mainly occurs in coastal habitats and occasionally spreads to riverine grasslands and grasslands along motorways, *T. repens* has a much wider ecological niche and occurs in a wide variety of grassland habitats and roadside verges (Huber and During 2000). For the present experiment, we collected 20–29 clonal fragments consisting of one unbranched stolon with 5–10 ramets in four grasslands where both species co-occurred, namely in two coastal dune slack and two riverine grasslands in The Netherlands (Oost-Voorne (51°85′N, 4°07′E) and Goedereede (51°84′N, 3°99′E), and Waardenburg (51°83′N, 5°28′E) and Pannerdensche Kop (51°89′N, 6°02′E), respectively). The distance between sampled plants was at least 1–2 m. These coastal dune slack and riverine habitats differ greatly in the frequency and type of disturbance. While the dune slack habitats are mainly characterized by flooding during winter and early spring caused by slowly rising water levels, the riverine grasslands can be flooded for several shorter times during the year but with faster inundation and higher final water tables. The latter situation is considered to cause greater physical disturbance.

**Figure 1.**
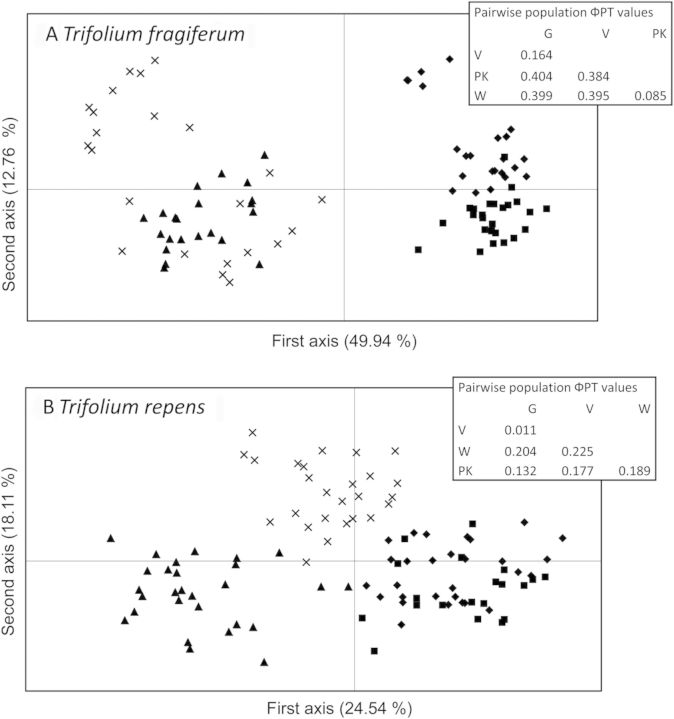
Principal coordinates analysis and ΦPT values computed from AMOVA on individuals of *T. fragiferum* (A) and *T. repens* (B) populations. Principal coordinates analysis plots and ΦPT values are based on 63 AFLP markers for *T. fragiferum* and on 47 AFLP markers for *T. repens*. The symbols represent riverine (filled uptriangle, Pannerdensche Kop, PK; cross, Waardenburg, W) and coastal dune slack (filled square, Oost-Voorne, V; filled diamond, Goedereede, G) populations. Percentages of total variance explained by each axis are noted in brackets. The inlaid box shows the ΦPT values for the pairwise population comparisons. Higher ΦPT values indicate a higher degree of differentiation between populations.

After collection, the plants were grown under homogeneous conditions in a research garden in containers (l × w × h: 60 × 40 × 30 cm) filled with a mixture of sand and commercial potting compost for 5 years. Plants were watered regularly and moved to new trays at the end of each year to maintain healthy clones. Leaf samples were taken for genotyping with amplification fragment length polymorphism (AFLP) analysis (Vos *et al.* 1995), using four EcoRI, MseI primer combinations with three selective nucleotides per primer. Plants that showed identical scores for all AFLP markers were considered genetically identical. Such duplicate genotypes were removed and only genetically different plants were kept for further analyses. The AFLP analysis resulted in a dataset with 63 AFLP markers for a total of 94 *T. fragiferum* genotypes and 47 AFLP markers for 103 *T. repens* genotypes. Principal coordinates analysis (PCoA) and pairwise genetic differentiation (ΦPT) values computed from AMOVA were used to analyse the genetic differentiation among populations and habitats. These values revealed strong genetic differentiation between riverine and coastal populations for both *T. fragiferum* and *T. repens* (Fig. 1). Genetic differentiation also occurred between the two coastal populations of *T. fragiferum* and the two riverine populations of *T. repens* (Fig. 1, where higher ΦPT values indicate higher genetic differentiation). For the fragmentation experiment, 10 genetically different plants were selected per population.

### Experiment

**Figure 2.**
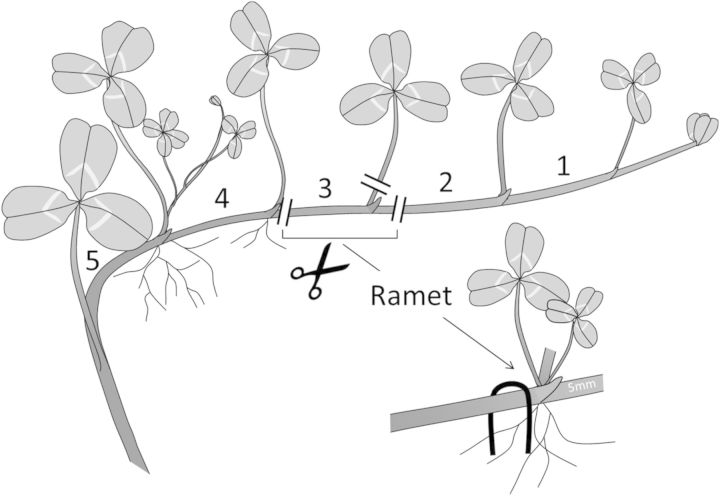
Illustration of how clonal fragments were created after the flooding treatment. Increasing numbers indicate developmental stages of the ramets; ramet 3 was selected for testing regrowth. Note that this ramet did not develop roots yet.

Prior to the experiment, clonal fragments of the genetically unique stock plants were moved to the greenhouse. Four clonal fragments per genotype were planted in individual trays filled with potting compost (Potgrond nr. 4; Lentse Potgrond, Lent, The Netherlands). After 6 weeks, the plants were large enough to start the experiment, each plant consisting of a complex clonal network, with ample first- and second-order branches. As a treatment prior to regrowth, two trays with plants of each genotype were flooded, while the other two trays were well watered but drained. During the flooding treatment, the water level was kept 2–3 cm above soil level to completely submerge the stolons and young leaves. After 2 weeks of flooding, which is sufficiently long to induce morphological responses linked to flooding adaptation and reflects typical flooding duration in riverine habitats in the growing period, the two original plants of each genotype were used to make a total of five apical cuttings per genotype × treatment combination. Owing to the fast developmental speed of the two species (growing an average of 2–3 ramets per week, even during soil flooding), these cuttings had been produced during the treatment period. Care was taken so that cuttings did not show developed root primordia or lateral branches. The third youngest ramet behind the apex from each apical cutting, consisting of an un-rooted node and its attached internode, was dissected and the leaf was removed to avoid excessive transpiration and hence water loss and to remove potentially confounding effects of leaf photosynthesis (Fig. 2). Stolon connections were severed immediately after the fourth ramet and 5 mm after the third ramet. We left the developmentally older internode attached, because resource transport predominantly takes place in the direction of the apex, leading to increased survival of ramets attached to the older internode (Dong *et al.* 2010). We did not sever the connection immediately after the ramet, because internodes often start to desiccate at the cut surface, which may cause damage to the meristem when being too close. The third ramet was thus attached to the whole developmentally older internode and 5 mm of the developmentally younger internode (Fig. 2). These cuttings will be referred to as clonal fragments in the remainder of the paper. After dissection, the length and diameter of the internode were determined with a digital caliper (type SKU 47257; Harbor Freight Tools, Camarillo, CA, USA) and a manual thickness meter (Dial Thickness Gage 2046F; Mitutoyo, Andover, UK), respectively. From these data the initial internode volume was calculated, which was used as a measure for internode size. The fragments were subsequently carefully placed in five trays (l × w × h: 60 × 40 × 15 cm) filled with moistened river sand and fixed to the substrate by a U-shaped plastic-coated wire. Each tray contained one replicate per genotype × treatment combination, and in total 160 fragments. The clonal fragments were arranged randomly in the trays. Thereafter, the trays were covered with a 1-cm-thick layer of plastic grains to achieve a high humidity around the clonal fragments and thus prevent desiccation of the plants. The trays were moved to a climate chamber with constant temperature and air humidity. The length of the light period was 16 h (photosynthetic photon flux density, 180–200 μmol PAR m^−2^ s^−1^; sodium lamps Philips HPS SON-T 600 W ﬂuorescent light and Philips TLD 58W/840R, both from Philips, Eindhoven, The Netherlands) and the night period was 8 h. Average temperatures were 21 °C during daytime and 19 °C during night-time with an average relative humidity of 60 %. Plants were carefully sprayed with water three times a week to prevent desiccation of the soil. After 2 weeks, the clonal fragments were carefully excavated and washed free of sand. For each clonal fragment it was determined whether the fragment was still alive, whether it had produced new leaves and roots, and if so, the number of leaves was counted. Plants were divided into the old internode, which was left attached to the plant after fragmentation, and the newly produced plant parts. Root and leaf tissue was not separated at harvest due to the small size of these new plant parts and because internode elongation had not yet started at the time of harvest. Root and leaf tissues were thus weighed together to establish plant weight. Biomass of the different clone compartments was determined after drying the plant parts at 70 °C for 72 h to constant weight. Occasionally, the clonal fragments had produced a flower. These fragments were noted as alive, but removed from further analyses, as meristems allocated to flowering cannot contribute to future plant growth (Huber and During 2000). The whole experiment was performed in two temporal blocks, which means that the whole procedure was repeated 3 weeks after the first block with the same genotypes. The whole experiment was comprised of 1600 clonal fragments, with 10 replicates per treatment and genotype combination.

### Data analyses

Survival was analysed by means of logistic regression, testing for the effects of species, habitat, treatment, population nested within habitat and genotype nested within population and habitat on ramet survival. Leaf number, stolon dimensions and biomass of the different compartments were analysed by means of three-way nested ANOVA, with species, habitat and treatment as the main factors, and the random factors population nested within habitat and genotype nested within population and habitat. A temporal block was added to the ANOVA model to account for differences between the two temporal blocks but not to the logistic regression. Whether or not initial internode volume affected survival and regrowth was analysed with linear regression analyses for each species×treatment×habitat combination separately. In order to be able to compare the magnitude of the effects of initial internode volume on regrowth between species and treatments and habitat of origin, we standardized the data prior to analyses following commonly used approaches to calculate standardized regression coefficients (Lande and Arnold 1983). Internode volume was standardized by subtracting the mean and dividing by the standard deviation. The performance parameters survival, leaf number and plant weight (excluding old stolon material) were relativized by dividing by the mean in order to be able to compare the different species and traits. Means and standard deviations were calculated for each species × treatment × habitat combination separately. As we assessed initial internode volume for each fragment separately, we were able to perform the regression analysis on the level of the individual plant. The regression analysis was performed for each treatment, species and habitat type separately, using the means and standard deviations for the respective groups. For each species and treatment, all plants originating from one habitat type were pooled for these regression analyses. The program package SAS (SAS 9.1; SAS Institute Inc., Cary, NC, USA) was used for all analyses.

## Results

### Initial internode dimensions

**Figure 3.**
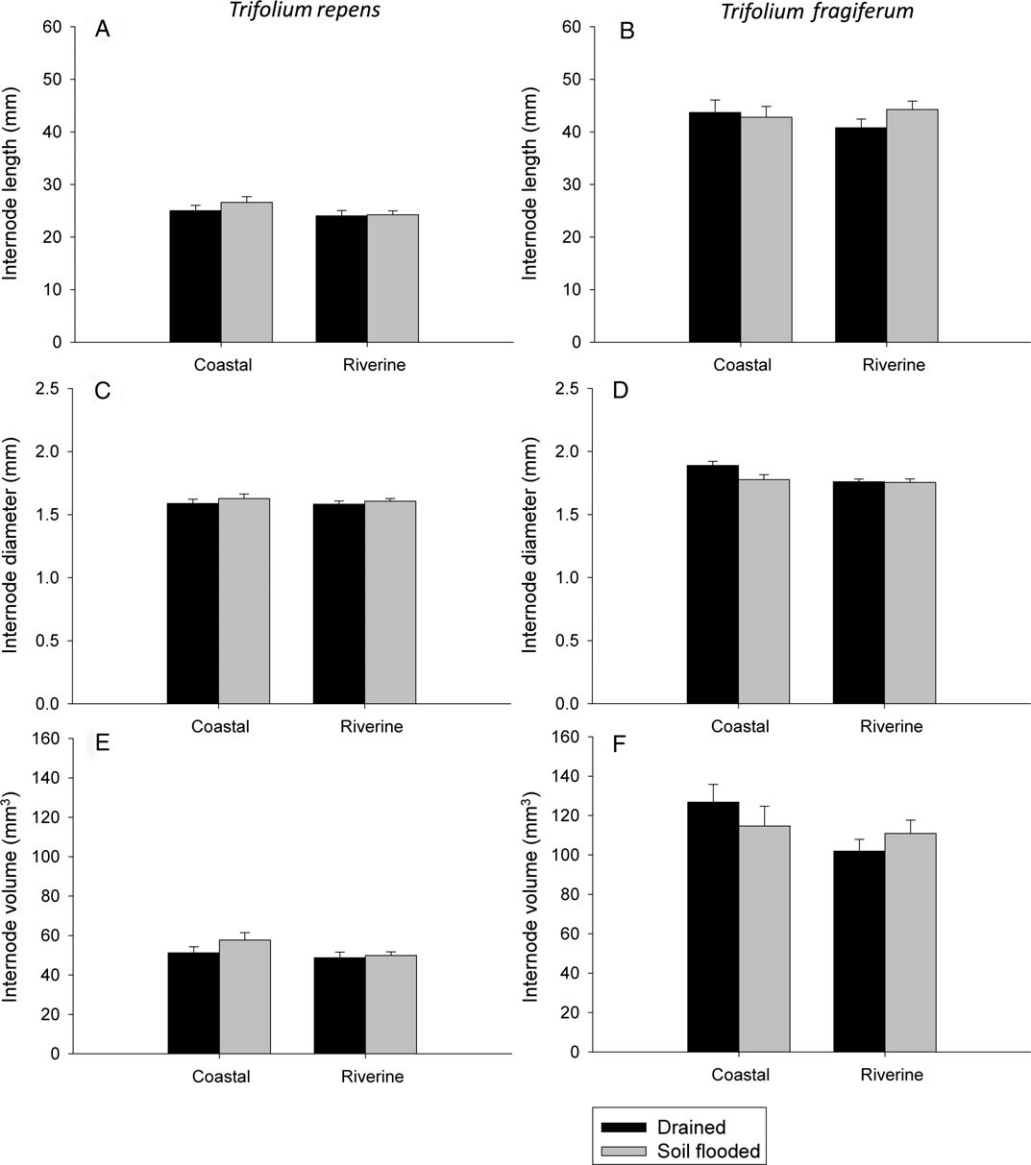
Characteristics of the attached internode after fragmentation (mean ± 1 SE) of plants that were previously subjected to drained (dark bars) or soil-flooded (light bars) conditions. Plants from the two populations within a given habitat of origin were pooled for the sake of clarity.

The two species differed conspicuously in internode dimensions. Generally, *T. repens* produced 50 % shorter and slightly (5–10 %) thinner internodes, resulting in lower internode volume than in *T. fragiferum* (Fig. 3, Table 1). There was no main effect of soil flooding on internode dimensions (Fig. 3, Table 1). Habitat, however, did affect this parameter to some degree. For *T. repen*s, internode volume did not differ between plants originating from the two different habitat types, while in *T. fragiferum* internodes in plants originating from coastal dune slacks were characterized by a 10–15 % higher volume than internodes in plants originating from riverine habitats. In *T. fragiferum*, the effect of soil flooding on internode volume differed between plants originating from coastal dune slacks and riverine habitats (*P* = 0.079 for the species × treatment × habitat interaction), as soil flooding decreased the internode volume of plants originating from coastal habitats by 10 % and increased the internode volume of plants originating from riverine habitats by 5 %. There was significant variation between populations within habitats and among genotypes within population for most internode characteristics (Table 1; P(H), Gen(S × P × H)). Genotypes also differed significantly in the response of internodes to soil flooding (Table 1, T × Gen(S × P × H), indicating a variation within populations for selection to act upon.

**Table 1.** Results from a four-way nested ANOVA testing for the effect of species (S; *T. repens* or *T. fragiferum*), treatment (T; soil flooded or drained), habitat of origin (H; riverine or coastal dune slacks), population (P) and genotype (Gen) on initial internode characteristics (length, diameter, volume), plant survival, leaf number, internode and plant weight at harvest. Survival was tested by means of logistic regression. *F*-values and their significance are given, except for survival where *χ*^2^ values and their significance are given. Only surviving plants were analysed for the data at harvest, resulting in lower df values (given in brackets). Significantly different values are indicated in bold, and marginally significant differences in italics. Significance levels are as follows: ***P ≤ 0.001, **0.001 < *P* ≤ 0.01, *0.01 < *P* ≤ 0.05, ^$^0.05 < *P* < 0.1, ns *P* ≥ 0.1.

	df	Internode length	Internode diameter	Internode volume	Survival	Leaf number	Internode weight	Plant weight
Species (S)	1,2	7426.3***	51.5*	6183.1***	*3.6*^$^	98.2**	60.0*	127.2**
Treatment (T)	1,2	1.1^ns^	0.5^ns^	0.1^ns^	5.4*	26.5*	*10.1*^$^	*16.6*^$^
S × T	1,2	0.0^ns^	4.7^ns^	1.8^ns^	0.1^ns^	2.0^ns^	3.4^ns^	2.1^ns^
Habitat (H)	1,2	0.3^ns^	43.4*	*9.3*^$^	0.1^ns^	8.3^ns^	0.2^ns^	*16.1*^$^
S × H	1,2	5.1^ns^	1.4^ns^	34.2*	2.2^ns^	3.3^ns^	0.3^ns^	0.0^ns^
T × H	1,2	0.5^ns^	1.3^ns^	0.8^ns^	0.1^ns^	0.2^ns^	0.0^ns^	1.6^ns^
S × T × H	1,2	0.3^ns^	2.2^ns^	*11.2*^$^	2.2^ns^	0.3^ns^	1.1^ns^	1.4^ns^
Population (habitat)	2, 1426 (646)	3.12*	0.5^ns^	3.2*	0.8^ns^	1.0^ns^	1.2^ns^	0.6^ns^
S × P(H)	2, 1426 (646)	0.2^ns^	8.1***	0.2^ns^	1.3^ns^	0.8^ns^	*2.6*^$^	0.2^ns^
T × P(H)	2, 1426 (646)	5.2**	4.4*	6.1**	0.4^ns^	0.6^ns^	4.6*	0.9^ns^
S × T × P(H)	2, 1426 (646)	6.0**	4.7*	1.2^ns^	2.4^ns^	1.0^ns^	4.7**	0.1^ns^
Gen(S × P × H)	72 (57), 1426 (646)	9.0***	8.1***	8.5***	32.93^ns^	1.2^ns^	4.1***	1.4*
T × Gen(S × P × H)	72 (57), 1426 (646)	2.2***	2.0***	2.9***	42.93^ns^	1.0^ns^	*1.3*^$^	0.8^ns^
Block	1, 1426 (646)	0.9^ns^	26.2***	1.5^ns^	–	0.6^ns^	4.7*	16.6***

### Response to fragmentation

**Figure 4.**
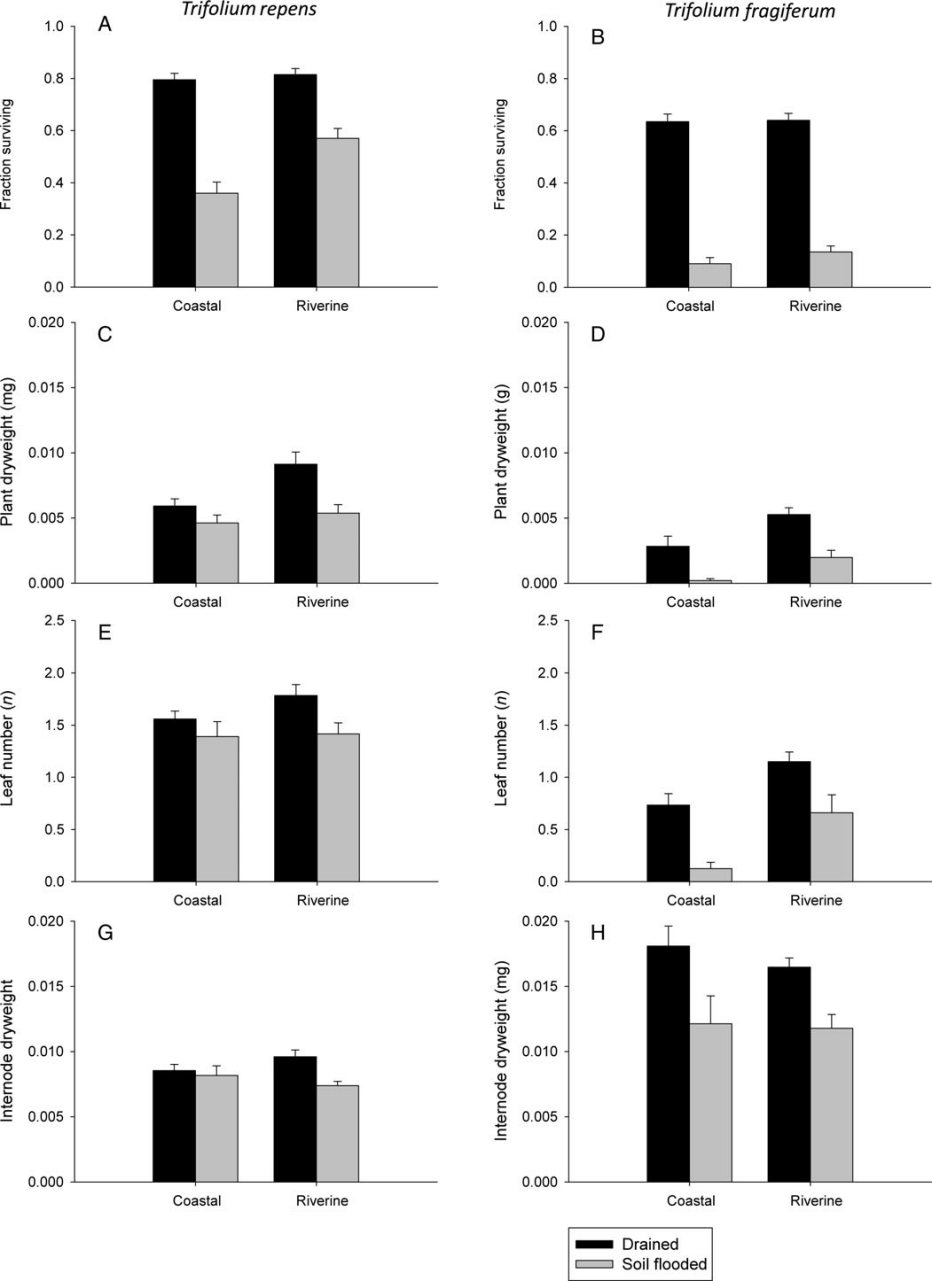
Plant growth and survival (mean ± 1 SE) 2 weeks after fragmentation. Plants were previously subjected to drained (dark bars) or soil-flooded (light bars) conditions. Plant weight indicates the biomass of new leaves and roots and excludes the original internode. Internode dry weight indicates the final dry weight of the internode that had remained attached to the plants after fragmentation. Plant weight, leaf number and internode weight are only given for those plants that actually survived. Plants from the two populations within a given habitat of origin were pooled for the sake of clarity.

Plant survival and regrowth (as determined by ramet weight and leaf number) after fragmentation differed between species and tended (*P* < 0.1) to be significantly affected by prior soil flooding. Overall, *T. fragiferum* showed lower survival ability than *T. repens* (Fig. 4A and B, Table 1). In all, 60–80 % of the drained plants survived clone fragmentation, whereas the survival ability of flooded plants dropped to 10 and 50 % for *T. fragiferum* and *T. repens*, respectively. The surviving plants had significantly more leaves and a higher dry weight in *T. repens* than in *T. fragiferum* (Fig. 4C–F, Table 1). Preceding soil flooding further reduced leaf number and plant dry weight in both species. Plants originating from riverine habitats produced 40–80 % more biomass after fragmentation than plants originating from coastal habitats, but this difference was only marginally significant statistically (*P* = 0.056; Fig. 4C and D, Table 1).

The internodes of drained plants of *T. fragiferum* were almost twice as heavy as those of drained *T. repens* plants (Fig. 4G and H, Table 1). Soil flooding decreased internode weight by up to 30 % in *T. fragiferum* and by 5–20 % in *T. repens* (*P* = 0.086).

There was no difference among genotypes with respect to survival and leaf production after fragmentation. However, plant weight (leaf and roots) and weight of the attached distal internode differed significantly among genotypes (Table 1).

### Relation between initial internode volume and survival and regrowth after fragmentation

Internode volume was correlated with survival and regrowth in clonal fragments, but the direction and strength of correlations were not consistent across species and treatments (Table 2). While initial internode volume was mainly negatively correlated with survival of plants subjected to drained conditions in *T. fragiferum*, it was positively correlated with survival in *T. repens*, if the plants had been subjected to soil flooding (Table 2). Initial internode volume was positively correlated with the biomass of newly produced ramets in *T. repens* as well as in *T. fragiferum* previously subjected to drained conditions.

**Table 2.** Correlation between internode volume and the performance parameters, i.e. survival, number of leaves and dry weight of the new plant (excluding the old internode). Standardized regression coefficients and their significance are given. Negative values indicate that performance parameters were negatively affected by increasing internode volume and positive values indicate that performance was positively affected by increasing internode volume. The analysis was performed for each species, treatment and habitat combination separately. Significantly different values are indicated in bold, and marginally significant differences in italics. Significance levels are as follows: ***P ≤ 0.001, **0.001 < *P* ≤ 0.01, *0.01 < *P* ≤ 0.05, ^$^0.05 < *P* < 0.1, ^ns^*P* ≥ 0.1.

	Survival	Leaf number	Plant weight
*Trifolium fragiferum*			
Coastal dune slack habitat, drained	−0.0137***	0.0138^ns^	0.0349*
Riverine habitat, drained	−0.0114**	*0.0140*^$^	0.0217*
Coastal dune slack habitat, flooded	−0.0004^ns^	−0.0140^ns^	0.0329^ns^
Riverine habitat, flooded	−0.0295*	−0.0039^ns^	−0.0086^ns^
*Trifolium repens*			
Coastal dune slack habitat, drained	−0.0057*	*0.0061*^$^	0.0250***
Riverine habitat, drained	0.0034^ns^	*0.0055*^$^	0.0161**
Coastal dune slack habitat, flooded	0.0258***	−0.0013^ns^	*0.0140*^$^
Riverine habitat, flooded	0.0098*	0.0086*	0.0231**

## Discussion

### Flooding reduces survival and regrowth in response to subsequent fragmentation

Plants in their natural environment are subjected to a multitude of environmental cues throughout their development, and conditions experienced at early developmental stages may affect response to subsequent cues (Weinig and Delph 2001; Bruce *et al.* 2007; von Wettberg *et al.* 2012). The effects may be direct if early conditions limit resource uptake and storage, or indirect through physiological changes such as to hormone balance or chemical composition of stored resources (Puijalon *et al.* 2008). Our results show that flooding strongly reduced survival and regrowth in response to subsequent fragmentation. This may have been due to reduced allocation of resources into storage in flooded plants, as under flooded conditions resources are, among others, allocated to morphological responses such as adventitious roots and increased leaf elongation (Huber *et al.* 2009; Chen *et al.* 2011). In addition, the cuticle of flooded leaves is often thinner, which makes the plants prone to desiccation as soon as the water recedes (Mommer *et al.* 2005, 2007; Chen *et al.* 2011). A similar process may have led to a reduction of cuticle thickness of submerged stolon internodes, thereby increasing water loss. Such water loss could explain the reduced survival of clonal fragments that were previously flooded in our experiment.

Fragmentation during natural flooding events may provide an important dispersal mechanism in clonal plants. It can thus be expected that genotypes characterized by reduced survival after flooding-induced fragmentation are selected against in highly disturbed riverine habitats. This is consistent with the relatively lower effects of flooding in the survival and regrowth in *T. repens*, a species common in such severely disturbed habitats, than in *T. fragiferum*, a species more common in less disturbed dune slack habitats.

### Habitat of origin hardly affects internode size and response to fragmentation

The type and frequency of flooding constitutes an important selection agent (Chen *et al.* 2011; Mony *et al.* 2011). We hypothesized that genotypes originating from disturbance-prone riverine habitats experience different selection pressures than genotypes from less disturbed dune slacks, leading to different resistances to fragmentation. Within species our experiment revealed only weak differences in internode dimensions and general resistance to fragmentation between genotypes originating from dune slack and riverine grasslands with the exception of the comparatively slower regrowth of coastal populations of *T. fragiferum* after fragmentation. However, while this habitat-induced difference was relatively large, it was only marginally significant statistically. This lack of differentiation, particularly in *T. repens*, may be due to relatively large gene flow. *Trifolium repens* is widely distributed and occurs in a wide variety of habitats, making it likely that seeds or clonal fragments disperse relatively easily among populations.

### Internode size contributes differently to plant survival and regrowth after fragmentation

Internode size is a trait subject to selection and the optimal size of internodes can depend on a multitude of environmental conditions (Hutchings *et al.* 1997; Weijschede *et al.* 2008). While internode length and diameter may be largely determined by optimal ramet spacing and morphological features associated with the transport function, increasing internode size may also positively affect the amount of resources stored in the internodes, and in turn increase survival and regrowth after clone fragmentation. Our current study tested the hypothesis that, comparable to results on the presence or absence of internodes (Stuefer and Huber 1999; Dong *et al.* 2010, 2012; Song *et al.* 2013), investment in larger internodes increases survival and regrowth after fragmentation. In contrast to this prediction, we found that survival probability markedly reduced with increasing internode size. The two species used in this experiment had a similar growth form and developmental rate. However, *T. fragiferum* produced internodes that were almost twice as long with an on average 2.5 times greater volume than internodes produced by *T. repens*. Despite this higher investment in internodes, *T. fragiferum* could not cope well with fragmentation, as fragmented plants had a much lower survival rate compared with *T. repens*. Generally, *T. repens* has a much broader distribution and ecological niche than *T. fragiferum* and can also occur in severely disturbed habitats. *Trifolium repens* may thus have evolved a high resistance to physical disturbance. Even under the extreme fragmentation regime applied in our experiment, close to 100 % of the fragments survived and continued growth in several genotypes of *T. repens*, indicating the great capacity of this species to establish even in strongly disturbed habitats.

Interestingly, comparison within species revealed that internode volume was negatively correlated with survival for *T. fragiferum*, indicating again that larger internodes may not be unequivocally beneficial in disturbed habitats. This result is surprising, as it contradicts the generally accepted notion that higher resource storage increases resistance to fragmentation (Suzuki and Stuefer 1999; Huber *et al.* 2004; Dong *et al.* 2010). Unfortunately, we cannot provide an unambiguous explanation of these surprising results. Several factors may have contributed to the decreased survival in plants with larger internodes. Carbohydrates stored in internodes may have attracted pathogens leading to a decay of the clonal fragment, an effect that is likely to be stronger in larger internodes. This interpretation is also supported by the fact that in previously flooded plants the effect of internode size was weaker. Flooded plants have probably invested less into resource storage in newly produced internodes, leading to the observed lower weight of internodes. Differences in desiccation after fragmentation may provide an alternative interpretation. Larger internodes were characterized by a higher surface-to-volume ratio, as variation in internode size was mainly due to variation in internode length. This may have led to higher evaporation in larger internodes and subsequent desiccation of the clonal fragment. Further research is needed to understand the specific mechanisms leading to reduced survival of larger clonal fragments.

It is, however, important to note that if plants were able to survive clonal fragmentation, the size of internodes was positively correlated with biomass gain after fragmentation. This is in line with results on a wide range of species (Dong *et al.* 2011; Song *et al.* 2013), since larger internodes are likely to contain more resources that can be reallocated to growth after fragmentation (Stuefer and Huber 1999). In line with this expectation, we found, in an unpublished experiment, that internode volume explains 47 % of the variation in carbohydrate content in the third youngest internode of *T. repens* subjected to control conditions (*n* = 15; *F* = 12,63; *P* = 0.003; J. Kassenberg and H. Huber, Radboud University, Nijmegen, The Netherlands, unpubl. res.). Our present results thus provide evidence for a more complex role of internode size in the ability of plants to withstand severe disturbance. While producing larger internodes may lower survival probability, it contributes positively to future growth and performance. This indicates that there may be contrasting selection pressures on internode size in stoloniferous species growing in severely disturbed habitats.

## Contributions by the Authors

H.H., E.J.W.V. and J.L.P. designed the experiments and wrote the paper, and H.H., E.J.W.V., J.L.P. and G.C. performed the experiments and analyses.

## Conflicts of Interest Statement

None declared.
